# Concurrent chronic follicular conjunctivitis and conjunctival MALT lymphoma potentially caused by *Chlamydia pneumoniae*

**DOI:** 10.1016/j.idcr.2025.e02251

**Published:** 2025-05-08

**Authors:** Yueh-Ling Chen, Shih-Ming Jung, Ching-Hsi Hsiao

**Affiliations:** a Department of Ophthalmology, Chang Gung Memorial Hospital, Linkou, Taiwan; b Department of Pathology, Chang Gung Memorial Hospital, Linkou, Taiwan; c College of Medicine, Chang Gung University, Taoyuan, Taiwan

**Keywords:** *Chlamydia*, Follicular conjunctivitis, Mucosa-associated lymphoid tissue (MALT) lymphoma

## Abstract

*Chlamydia*, particularly *C. psittaci*, has been implicated in the pathogenesis of ocular adnexal lymphoma with varying geographical prevalence. We report a patient with concurrent chronic follicular conjunctivitis and conjunctiva mucosa-associated lymphoid tissue (MALT) lymphoma, potentially caused by *C. pneumoniae.* A 30-year-old female presented with a 10-month history of persistent discharge from the left eye. Examination of the conjunctiva revealed large follicles and a salmon-patched lesion over inferior fornix. Chlamydial antigen testing from conjunctival swab was positive, so the patient underwent a 3-week course of oral doxycycline (100 mg, twice daily), after which the discharge resolved, but the conjunctival mass persisted. A subsequent conjunctival biopsy identified extranodal marginal zone lymphoma of MALT type. Following systemic evaluations, clinical staging indicated T1N0M0, stage IE. The patient received a total 3060 cGy of radiotherapy in 17 fractions, leading to regression of the lesion. Touchdown Enzyme Time-Release polymerase chain reaction analysis of formalin-fixed paraffin-embedded biopsy samples confirmed the presence of *C. pneumoniae* DNA. This case highlights a potential association between chronic follicular conjunctivitis and conjunctival MALT lymphoma, possibly linked to *C. pneumoniae* infection. The potential causality warrants further investigation.

## Introduction

Adult inclusion conjunctivitis is a sexually transmitted disease prevalent among young adults. It typically presents unilaterally but may involve both eyes. Patients often experience mild symptoms persisting for weeks to months. The clinical features include enlarged preauricular lymph nodes, conjunctival follicles, punctate epithelial keratitis, intraepithelial keratitis, and subepithelial corneal opacities [1]. The pathology demonstrates intranuclear inclusions in plasma cells [1].

Ocular adnexal lymphomas (OALs), which affects lacrimal gland, lids, the orbit and the conjunctiva, are a heterogeneous group of malignancies, accounting for about 1–2 % of all non-Hodgkins lymphomas, and 8 % extranodal lymphoma [2]. Nearly all OALs are of B-cell phenotype, with extranodal marginal zone lymphoma of mucosa-associated lymphoid tissue (MALT) type being the most common subtype, accounting for 35–90 % of cases. Other subtypes include follicular lymphoma, diffuse large B-cell lymphoma, and mantle cell lymphoma [3]. Chronic antigenic stimulation, such as autoimmune disease and chronic infections, is believed to be the main etiological factor for OALs, with *Chlamydia*, particularly *C. psittaci*, being notably associated [4], [5], [6], [7].

Herein, we presented a case of concurrent chronic follicular conjunctivitis and conjunctival MALT lymphoma, potentially caused by *C. pneumoniae.*

## Case presentation

**Fig. 1:**
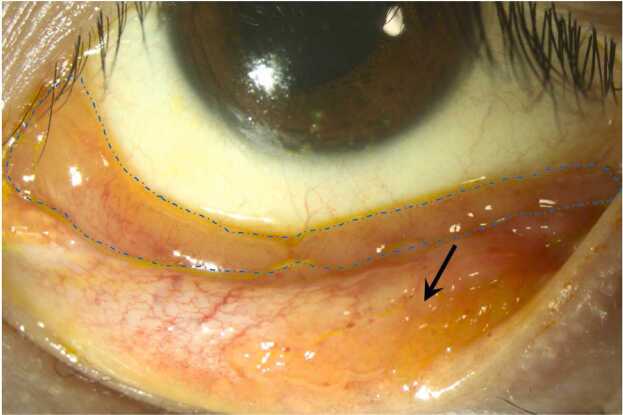
The slit-lamp photograph of left eye of the patient showing large follicles over palpebral conjunctiva (arrow), and a conjunctival mass occupied the entire inferior fornix (blue dashed lines).

A 30-year-old female patient was referred to our clinic with persistent discharge in the left eye for 10 months. She had a major traffic accident with intracranial hemorrhage and multiple skull fractures one and a half years ago but recovered remarkably well after surgery and rehabilitation. She reported no prior ophthalmological treatment, history of pneumonia, or allergies. On examination, corrected visual acuity was 20/20, and intraocular pressure measured 19 mmHg in both eyes. Slit-lamp examination revealed large follicles on the inferior palpebral conjunctiva and a salmon-patched lesion in the inferior fornix of the left eye (Fig. 1). Evaluation of the cornea, anterior chamber, lens, and fundi showed no abnormalities. She denied respiratory symptoms, pet exposure, and urinary complaints, but mild vaginal discharge was noted.

**Fig. 2:**
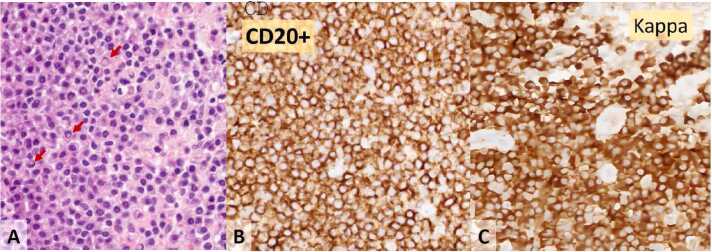
The stains of left eye conjunctival biopsy showing Dutcher bodies (arrows, A) in plasma cells and lymphocytes positive for CD20 (B) and κ light chain (C).

Based on the history and ocular findings, a preliminary diagnosis of adult inclusion conjunctivitis in the left eye was made. A conjunctival swab was taken for chlamydial antigen testing, which later returned positive. She was prescribed a three-week course of oral doxycycline 100 mg twice daily. Although her symptoms, such as discharge, resolved following treatment, the conjunctival mass persisted. Subsequently, a conjunctival biopsy was performed, revealing extranodal marginal zone lymphoma of MALT type. Pathological examination depicted scattered germinal centers surrounded by small lymphoid tumor cells, with several Dutcher bodies noted in plasma cells (Fig. 2A); CD20-positive B cells were observed (Fig. 2B), and there was a predominance of plasma cells with kappa light chains (Fig. 2C), indicating light chain restriction.

Systemic evaluations, including a comprehensive physical examination, complete blood count, and biochemical screening, were conducted by a hematologist. Leukocytosis (12,100/µL) with a normal differential count was the only abnormal laboratory finding. Computed tomography (CT) scans showed no evidence of lymphoma in other organs. Clinical staging suggested T1N0M0, stage IE. The patient underwent radiotherapy, receiving a total dose of 3060 cGy in 17 fractions targeting the upper and lower eyelids, underlying conjunctiva, and lacrimal gland in the left eye. The lesion completely resolved, with the sequela of dry eye and cataract with best corrected VA 20/50 in the left eye at 4-year follow-up.

**Fig. 3:**
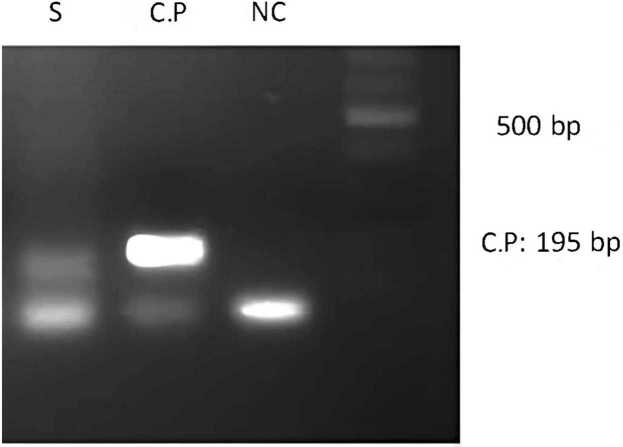
The PCR product showing positive for *Chlamydia pneumoniae*. S: sample, C.P.: *Chlamydia pneumoniae*, NC: negative control.

We got formalin-fixed paraffin-embedded biopsy samples of the patient. Following dewaxing and DNA extraction, Touchdown Enzyme Time-release polymerase chain reaction (PCR) was performed to detect *C. psittaci, C. pneumoniae*, and *C. trachomatis*. The PCR results revealed the presence of *C. pneumoniae* DNA (Fig. 3).

## Discussion

In this case report, chronic follicular conjunctivitis was observed concurrently with conjunctival MALT lymphoma. Chlamydial conjunctivitis was confirmed by a chlamydial antigen testing, while the presence of *C. pneumoniae* DNA was detected in the specimens of conjunctival MALT lymphoma. Hence, it suggests a potential link between chronic follicular conjunctivitis and conjunctival MALT lymphoma, with a possible association with *C. pneumoniae* in this patient.

In this patient, the initial diagnosis of adult inclusion conjunctivitis was made based on persistent conjunctival discharge, mild vaginal discharge, the presence of conjunctival follicles, and confirmed by a positive chlamydial antigen testing. In addition, the symptoms responded well to oral doxycycline. Adult chlamydial conjunctivitis is a sexually transmitted disease, often associated with chlamydial urethritis or cervicitis, and manifests with a wide range of clinical symptoms including mucopurulent discharge, foreign body sensation, redness, tearing, photophobia, and hyperemia of palpebral conjunctiva. Diagnosis is based on Giemsa staining of conjunctival smears, antigen detection assays such as direct fluorescent antibody and enzyme immunoassay, culture isolation in McCoy cells and PCR assay [1]. Treatment options included tetracycline, erythromycin, and sulfonamides, and systemic antibiotic is strongly recommended to mitigate the risk of reinfection [1]. Genital strains of *C. trachomatis* (serovars D–K) are responsible for adult inclusion conjunctivitis [1]. Unlike *C. trachomatis*, *C. pneumoniae* is typically not associated with sexually transmitted infections and is more commonly linked to respiratory tract infections. Interestingly, Lietman et al. used PCR and automatic sequencing to identify the presence of *C. psittaci,* and *C. pneumoniae* DNA in the conjunctival swabs from some patients with chronic follicular conjunctivitis [8]. Our patient had no respiratory symptoms. The mild vaginal discharge—reported only after directed questioning and not considered significant by the patient—could be an incidental finding unrelated to the ocular condition.

In this patient, the pathological report of the conjunctival mass was MALT lymphoma, and the presence of *C. pneumoniae* DNA was detected by PCR method. It is well known that some lymphoma entities are associated with chronic infections that probably drive the first steps towards malignant B-cell transformation through a sustained stimulation of the immune system. The best-documented bacterium-lymphoma association is observed in gastric marginal zone B cell lymphoma resulting from chronic local *Helicobacter pylori* infection, supported by epidemiological, histopathological, molecular, and therapeutic evidence [9]. *Chlamydia*, a kind of obligate intracellular bacteria, are implicated in various human diseases and potentially contribute to tumor development. Ferreri et al. from Italy first reported that *C. psittaci*, a known etiologic agent of psittacosis, was present in 80 % of OALs [4] and eradication of the organism by antibiotic treatment led to partial or complete regression of the disease in majority of the cases [5]. Subsequent studies revealed variable prevalence of *C. psittaci* infection in OALs across different regions. Additionally, *C. trachomatis* and *C. pneumoniae* were also implicated in OAL pathogenesis, with varying prevalence geographically. An updated systematic review and meta-analysis, incorporating 37 studies on 1188 OALs, highlighted highly variable *C. psittaci* involvement in OALs across countries, with Italy and Korea showing the highest prevalence (> 50 %). Other *Chlamydia* species, such as *C. pneumoniae* (14.7 % in China) and *C. trachomatis* (25.7 % in the United Kingdom), are more prevalent in OAL patients in some regions; *Chlamydia* infection is significantly more common in MALT-type than non-MALT OALs [7]. Although the association between OAL and *Chlamydia* species is established in some regions, few studies have directly linked conjunctivitis with OALs. Yeung et al. reported an 18-year-old man diagnosed with adult inclusion conjunctivitis and conjunctival MALT lymphoma; the former was confirmed by chlamydial antigen testing, while the latter was not tested for *Chlamydia* [10]. Ferreri et al. were the only ones to show that a significant proportion of patients with MALT OALs had *C. psittaci* in their conjunctival swabs; these patients also more often reported prolonged contact with household animals and chronic conjunctivitis, suggesting an increased risk of *C. psittaci* exposure [6]. Our case may further support the link between chlamydial conjunctivitis and MALT lymphoma.

Radiotherapy remains the mainstay of treatment of OALs, although it may cause some side effects such as cataracts, dry eyes, retinopathy, or corneal ulcers, as observed in our patient. Alternatively, antibiotics can be considered as another option to treat MALT lymphoma. For example, bacterial eradication therapy is commonly used to treat gastric MALT lymphomas associated with *H. pylori* infection [9].

Similarly, doxycycline therapy has shown tumor regression in 38 % of ocular adnexal MALT lymphoma cases [5]. However, in our patient, the conjunctival tumor did not regress after a 3-week course of doxycycline treatment. Generally speaking, ocular adnexal MALT lymphoma has a favorable response to treatment. The systemic dissemination rate is 5–10 %, and only about 5 % of patients die from the disease. The 5-year survival rate exceeds 90 %, particularly when the disease primarily involves the conjunctiva, indicating a favorable prognosis with appropriate treatment [11].

Adult inclusion conjunctivitis, a prevalent sexually transmitted disease, is primarily caused by *Chlamydia trachomatis*. Meanwhile, MALT lymphoma, the most common type of OALs, has been hypothesized to develop from chronic inflammation such as chlamydial infections. In this patient, chronic follicular conjunctivitis has been found to concur with conjunctival MALT lymphoma, with *Chlamydia pneumoniae* DNA being detected. While the causal relationship warrants further investigation, this case underscores the importance of considering lymphoproliferative disorders in cases of chronic conjunctivitis and highlights the need for comprehensive evaluation and management strategies in such situations.

## CRediT authorship contribution statement

**Jung Shih-Ming:** Methodology, Formal analysis. **Chen Yueh-Ling:** Writing – original draft, Investigation, Data curation. **Hsiao Ching-Hsi:** Writing – review & editing, Supervision, Methodology, Investigation, Funding acquisition, Conceptualization.

## Ethical approval and informed consent statements

This study was approved by the Institutional Review Board of Chang Gung Memorial Hospital, Taiwan (202200489). The consent form for using the medical information was obtained from the patient.

## Ethical approval

This study was approved by the Institutional Review Board of Chang Gung Memorial Hospital, Taiwan (IRB no. 202200489).

## Consent

Written informed consent was obtained from the patient for publication of this case report and accompanying images. A copy of the signed consent is available for review by the Editor-in-Chief upon request.

## Funding

This study was supported by 10.13039/100012553Chang Gung Memorial Hospital, Taiwan (CMRPG1M0061). The funding organizations had no role in the design or conduct of the study.

## Declaration of Competing Interest

The authors declare that they have no known competing financial interests or personal relationships that could have appeared to influence the work reported in this paper.

## Data Availability

The data of this study are available from the corresponding author, Ching-Hsi Hsiao, on reasonable request.

## References

[1] Satpathy G., Behera H.S., Ahmed N.H. (2017). Chlamydial eye infections: current perspectives. Indian J Ophthalmol.

[2] Stefanovic A., Lossos I.S. (2009). Extranodal marginal zone lymphoma of the ocular adnexa. Blood.

[3] Rosado M.F., Byrne G.E., Ding F., Fields K.A., Ruiz P., Dubovy S.R., Walker G.R., Markoe A., Lossos I.S. (2006). Ocular adnexal lymphoma: a clinicopathologic study of a large cohort of patients with no evidence for an association with Chlamydia psittaci. Blood.

[4] Ferreri A.J., Guidoboni M., Ponzoni M., De Conciliis C., Dell'Oro S., Fleischhauer K., Caggiari L., Lettini A.A., Dal Cin E., Ieri R. (2004). Evidence for an association between Chlamydia psittaci and ocular adnexal lymphomas. J Natl Cancer Inst.

[5] Ferreri A.J., Ponzoni M., Guidoboni M., De Conciliis C., Resti A.G., Mazzi B., Lettini A.A., Demeter J., Dell'Oro S., Doglioni C. (2005). Regression of ocular adnexal lymphoma after Chlamydia psittaci-eradicating antibiotic therapy. J Clin Oncol.

[6] Ferreri A.J., Dolcetti R., Dognini G.P., Malabarba L., Vicari N., Pasini E., Ponzoni M., Cangi M.G., Pecciarini L., Resti A.G. (2008). Chlamydophila psittaci is viable and infectious in the conjunctiva and peripheral blood of patients with ocular adnexal lymphoma: results of a single-center prospective case-control study. Int J Cancer.

[7] Travaglino A., Pace M., Varricchio S., Della Pepa R., Iuliano A., Picardi M., Pane F., Staibano S., Mascolo M. (2020). Prevalence of Chlamydia psittaci, Chlamydia pneumoniae, and Chlamydia trachomatis determined by molecular testing in ocular adnexa lymphoma specimens. Am J Clin Pathol.

[8] Lietman T., Brooks D., Moncada J., Schachter J., Dawson C., Dean D. (1998). Chronic follicular conjunctivitis associated with Chlamydia psittaci or Chlamydia pneumoniae. Clin Infect Dis: Publ Infect Dis Soc Am.

[9] Zucca E., Bertoni F., Roggero E., Bosshard G., Cazzaniga G., Pedrinis E., Biondi A., Cavalli F. (1998). Molecular analysis of the progression from Helicobacter pylori-associated chronic gastritis to mucosa-associated lymphoid-tissue lymphoma of the stomach. N Engl J Med.

[10] Yeung L., Tsao Y.P., Chen P.Y., Kuo T.T., Lin K.K., Lai L.J. (2004). Combination of adult inclusion conjunctivitis and mucosa-associated lymphoid tissue (MALT) lymphoma in a young adult. Cornea.

[11] Chung H.U., Son J.H. (2022). Ocular adnexal mucosa-associated lymphoid tissue lymphoma: a narrative review. J Yeungnam Med Sci.

